# Paraoxonase-1 55 LL Genotype Is Associated with No ST-Elevation Myocardial Infarction and with High Levels of Myoglobin

**DOI:** 10.1155/2012/601796

**Published:** 2012-03-25

**Authors:** Francesca Marchegiani, Liana Spazzafumo, Maurizio Cardelli, Mauro Provinciali, Francesco Lescai, Claudio Franceschi, Roberto Antonicelli

**Affiliations:** ^1^Advanced Technology Centre for Aging Research, Italian National Research Centre on Aging (INRCA), INRCA-IRCCS, 60121 Ancona, Italy; ^2^Centre of Biostatistics, Italian National Research Centre on Aging (INRCA), 60121 Ancona, Italy; ^3^Institute of Child Health, University College London, London, UK; ^4^Department of Experimental Pathology, University of Bologna, Bologna, Italy; ^5^Interdipartimental Centre “L. Galvani”, University of Bologna, Bologna, Italy; ^6^Department of Cardiology (CCU), Italian National Research Centre on Aging (INRCA), 60121 Ancona, Italy

## Abstract

It is well known that serum paraoxonase (PON1) plays an important role in the protection of LDL from oxidation. PON1 55 polymorphism is currently investigated for its possible involvement in cardiovascular diseases. The objective of our study is to verify if PON1 55 polymorphism is associated with risk of acute coronary syndrome (ACS) and with biochemical myocardial ischemia markers, such as troponin I, creatine kinase (CK)-MB, myoglobin, and C-reactive protein. We analysed PON1 55 polymorphism in a total of 440 elderly patients who underwent an ACS episode: 98 patients affected by unstable angina (UA), 207 AMI (acute myocardial infarction) patients affected by STEMI (ST elevation), and 135 AMI patients affected by NSTEMI (no ST elevation). We found that individuals carrying PON1 55 LL genotype are significantly more represented among AMI patients affected by NSTEMI; moreover, the patients carrying LL genotype showed significantly higher levels of myoglobin in comparison to LM + MM carriers patients. Our study suggests that PON1 55 polymorphism could play a role in the pathogenesis of cardiac ischemic damage. In particular, the significant association between PON1 55 LL genotype and the occurrence of a NSTEMI may contribute to improve the stratification of the cardiovascular risk within a population.

## 1. Introduction

In western developed countries the atherosclerosis-related diseases, such as cardiovascular disease (CVD), remains so far the major cause of morbidity and premature death [1]. For this reason, atherosclerotic cardiovascular disease represents a major public health concern, and many efforts are addressed to better understand the mechanisms underlying this important pathology. The oxidative modification of low-density lipoprotein (LDL) in the arterial wall is claimed to play a central role in the pathogenesis of atherosclerosis. The oxidative stress is demonstrated to increase the formation of oxidized macrophages which are, in turn, responsible for the oxidation of LDL. Early studies focused on atherosclerosis generally considered LDL as the main cause of this pathology, and the inclusion of statins in clinical practice has significantly improved the quality of life of patients. The hypothesis of the inflammation as an underlying cause of atherosclerosis has been proposed only recently. For a long time, these two aspects (lipids and inflammation) have been kept apart, but actually they are the two faces of the same medal and both should be considered when we deal with atherosclerosis. Serum paraoxonase (PON1), an HDL-associated enzyme, plays an important role in the protection of LDL from oxidation, and it may attenuate the development of atherosclerosis [2, 3]. In fact, PON1 hydrolyzes and reduces lipid peroxides in lipoproteins and in arterial cells. The atheroprotective function of PON1 has been also demonstrated in PON1 knock-out mice, which exhibited an accelerated atherosclerosis in contrast to hPON1 transgenic mice where the lesion size was decreased [4]. PON1 exerts its anti-inflammatory properties mainly hydrolyzing hydrogen peroxide, a major reactive oxygen species produced under conditions of inflammation. The most studied PON1 gene polymorphisms are due to amino acid substitutions at position 192 (Gln-Arg) and at position 55 (Leu-Met) in the coding region of the gene. Alleles at codon 192 (Q and R alleles) and 55 (L and M alleles) in PON1 locus have been associated with enzymatic activity and concentration, respectively [5–7]. PON1 192 and 55 polymorphisms have been widely investigated especially for their possible involvement in the onset or severity of cardiovascular disease (CVD) [8]. Some studies have found these two polymorphisms associated with the risk of cardiovascular diseases, but others have reported no association [8]. Until now, a definitive response about the role of PON1 genetic polymorphisms carried out in CVD does not exist, and further studies are necessary to better clarify the real involvement of this gene in cardiovascular pathology. The purpose of this study is to better explore this issue analysing the relationship between PON1 55 polymorphism and ACS (acute coronary syndrome). Today, more than ever, the use of genetics in the formulation of a diagnosis is a daily need. In fact, it is widely demonstrated that the traditional resources available to the clinician, that is, international guidelines and cardiac enzymes, are insufficient, and this is especially true in elderly patients where, frequently, the cardiovascular disease has an unusual onset, often leading to misdiagnosis. Therefore, studies carried out to find new molecular markers are warranted.

## 2. Materials and Methods

### 2.1. Sample

The sample enrolled for this study was composed of 440 elderly patients affected by ACS (acute coronary syndrome) (246 males and 194 females, mean age: 79.28 ± 9.94 years) consecutively admitted to the Coronary Care Unit (CCU) of the Italian National Research Center on Aging (INRCA) in a consequence of an ACS. Ninety-eight patients were affected by unstable angina (UA), two hundred and seven patients were affected by STEMI (ST-elevation myocardial infarction) and one hundred and thirty-five patients were affected by NSTEMI (no ST-elevation myocardial infarction). All patients were considered eligible if they fulfilled the diagnostic criteria for ACS [9]. Exclusion criteria were severe anemia, cancer, or life expectancy less than 12 months for other severe illness. Patients were then included in a one-year followup aimed at detecting the cardiovascular mortality rate (complete followup was available in 67.3% of the patients, *n* = 296).

 During the hospitalisation the AMI diagnosis was confirmed with instrumental examination, such as coronary angiography and 2D-ecocardiography. The patients enrolled in the study were stratified as patients with a previous history of CHD, if the presence of CHD was previously documented, or patients without a history of CHD, if the current ACS episode was the first CHD manifestation. All subjects gave their informed consent to the study, which was approved by the Ethics committee of the INRCA. In all patients we evaluated arterial blood pressure, total and HDL-cholesterol, C-reactive protein (CRP), MB fraction of creatine kinase, measured by mass assay (CK-MB), troponin I (TnI), and myoglobin levels. Levels of HDL cholesterol were defined as low if <30 mg/dL (0.78 mmol/L), and hypercholesterolemia was defined by 200 mg/dL (5.18 mmol/L) cholesterol levels according to WHO guideline [10]. The diagnosis of arterial hypertension was made according to the 2003 European Society of Hypertension-European Society of Cardiology Guidelines for the management of arterial hypertension [10], if office blood pressure was repeatedly >140/90 mmHg or if patients were under antihypertensive treatment. Diagnosis of type 2 diabetes mellitus was based on revised American Diabetes Association diagnostic criteria [11].

### 2.2. Genotyping

DNA was extracted from peripheral blood mononuclear cells (PBMCs) using phenol/chloroform, according to standard procedures [12]. Polymerase chain reactions were performed using primer sequences derived from published data and specific for the amplification of the regions surrounding codon 55. The amplification reactions and methodologies have been previously described [13].

### 2.3. Statistical Analysis

Arterial hypertension, type 2 diabetes mellitus, documented history of CHD, hypercholesterolemia and low HDL cholesterol were coded as binary variables on the basis of the presence or absence of these conditions. Smoking habit was assessed through a questionnaire, and each subject was coded as current or noncurrent smoker (never and ex-smoker). C-reactive protein (CRP), MB fraction of creatine kinase (CK-MB), troponin, and myoglobin levels were natural log transformed before statistical analysis to achieve a normal distribution. Differences for categorical and continuous variables were assessed by *χ* ^2^ test and unpaired *t*-test, respectively. PON1 55 genotypes were grouped and analyzed as M+ (LM + MM genotypes) and M*‒* (LL genotype) carriers.

Because diagnoses are defined as 3-level categorical outcome (UA, STEMI, and NSTEMI), the multinomial logistic regression was performed to evaluate the relationships between the ACS and PON1 55 carriers. Odds ratio (OR) and 95% confidence interval (95% CI) were estimated for the STEMI and NSTEMI groups with respect to UA patients (reference group).

The hazard ratios (HRs) with corresponding 95% confidence interval (CI) for the occurrence of one-year follow-up mortality associated with the following covariates (PON1 55 carriers, age, documented history of CHD, arterial hypertension, smoking habit, hypercholesterolemia, type 2 diabetes mellitus, low HDL cholesterol, ACS diagnoses, CRP, troponin, CK-MB, and myoglobin levels) were estimated with Cox proportional hazards analysis.

Throughout the study the level of statistical significance was defined by a two-tailed *P* value <0.05. All the analyses were performed by tests implemented with the SPSS package for Windows, version 18 (SPSS, Chicago, Illinois, USA).

## 3. Results

The baseline clinical characteristics of 440 CHD elderly patients affected by an ACS are reported in Table 1. PON1 55 carriers frequency distributions, separately considered on the basis of the diagnosis of UA, STEMI, and NSTEMI are reported in Table 2. Significant difference among the three groups of ACS was evident when carrier frequency distribution was compared (*χ* ^2^ = 6.158, d.f. = 2 *P* = 0.046 for carrier frequency). In particular, by the multinomial logistic regression model, we found that subjects carrying LL genotype (M*‒* carriers) have a higher risk to develop a NSTEMI, in respect to the other ACS groups (OR: 1.855, 95% CI, 1.090 to 3.156; *P* = 0.023; UA patients were considered as the reference group). We then assessed the mean plasma levels of C-reactive protein and myocardial ischemia markers (troponin I, CK-MB, and myoglobin) according to the PON1 55 carriers. We found that among ACS patients, those who were carriers of the PON1 55 LL genotype displayed significantly higher plasma levels of myoglobin (Table 3). Interestingly, also for the other biochemical parameters considered (CRP, troponin I, and CK-MB), we found that LL patients displayed higher levels in respect to M+ carriers, although not significant (Table 3).

The one-year follow-up mortality rate was not significantly associated with PON1 55 carriers neither with any specific ACS diagnoses, such as STEMI, NSTEMI, and UA, nor with history of CHD, smoking habits, hypercholesterolemia, arterial hypertension, CK-MB levels, or troponin I levels (data not shown). Conversely, a significant association with mortality rate was found for the following variables: age (HR: 1.060, 95% CI, 1.020 to 1.102; *P* = 0.003), history of type 2 diabetes mellitus (HR: 1.874, 95% CI, 1.104 to 3.183; *P* = 0.020), HDL cholesterol levels (HR: 1.720, 95% CI, 1.003 to 2.948; *P* = 0.049), C-reactive protein (HR: 1.302, 95% CI, 1.073 to 1.580; *P* = 0.008), and myoglobin levels (HR: 1.511, 95% CI, 1.151 to 1.983; *P* = 0.003). Moreover, PON1 55 M+ and M*‒* carriers did not significantly correlate with history of CHD, diabetes mellitus, arterial hypertension, smoking habits, hypercholesterolemia, and low HDL-cholesterol (data not shown).

## 4. Discussion

Atherosclerosis is a multifactorial disease of great complexity, and we are only now beginning to sort out the many elements involved especially in the later stages of the disease. Accordingly, the main aim of our study was to investigate the relationship between the PON1 55 polymorphism and acute coronary syndrome in elderly patients. In the past, we have extensively analyzed the role of paraoxonase-1 in healthy elderly people, that is, centenarians, both as genetics and as activity and mass of the protein (as reviewed in [14]). The first result that we have obtained in this study is that PON1 55 M*‒* carriers have a significantly different distribution in the three groups of patients, that is, UA, STEMI, and NSTEMI patients. In particular, subjects carrying LL genotype have almost double the risk to develop an AMI of NSTEMI type. The other important finding is that individuals carrying LL genotype displayed significantly higher plasma levels of myoglobin in respect to M+ carriers. Although not significantly, also troponin I, CK-MB, and CRP levels were higher in LL subjects. All of them are important biomarkers evaluated in case of patients manifesting the classical symptoms of an ACS episode. The fact that these biomarkers were higher in LL patients compared to those with LM or MM genotype could suggest that they will have to face a more severe ACS episode. This finding, to the best of our knowledge, has not yet been reported in an other study. In this work the absence of a control group inhibits us to state if PON1 55 polymorphism could be responsible for the onset of cardiovascular disease. We can only suggest that this polymorphism could modulate the pathogenesis and the seriousness of cardiovascular disease.

Based on the above-mentioned assumptions, we would expect that the mortality rate would be higher in LL patients in respect to the other (LM or MM patients). Surprisingly, the one-year follow-up mortality rate was influenced by other variables, such as age, history of type 2 diabetes mellitus, HDL-cholesterol levels, C-reactive protein levels, and myoglobin levels. The fact that PON1 55 polymorphism does not directly influence the mortality rate could depend by the fact that subjects with the followup are a subgroup, and perhaps too few, or that the genotype alone could not explain the entire phenomenon. However, it is very interesting to note that among the variables affecting the mortality rate we have found myoglobin levels and HDL-cholesterol levels. The latter is particularly interesting as paraoxonase is a high-density lipoprotein- (HDL-) associated esterase that hydrolyses lipoperoxides, and so it could play a role, albeit in an indirect way.

In the literature, the possible role played by PON1 55 polymorphism, and in particular LL genotype, in the onset of cardiovascular heart disease (CHD) was extensively investigated and several authors have found a positive association between this variant and the cardiovascular pathologies (reviewed in [8]).

Conversely, the number of the studies that do not consider the onset of the disease but its severity are much less numerous, at least until now. Perhaps, it depends by the fact that the genetics of an individual is considered an important characteristic to determine if the individual could be at risk of developing a disease in order to make primary prevention. However, actually, it could also be important once the disease has occurred to stratify the subjects and treat them differently according to the genetic background, focusing on those who are at increased risk (secondary prevention).

In a recent paper, the authors to find the possible candidate genes involved in the pathogenesis of coronary atherosclerosis analyzed 1936 tag SNPs within 116 candidate genes. Interestingly, they found three SNPs associated with the disease, and one of them (rs854563) is included in an intron in the same region of PON1 55 polymorphism [15]. Another paper analyzed PON1 192 and 55 polymorphisms in the etiology of CAD. The authors failed to find an association between PON1 55 polymorphism and the onset of cardiovascular disease, but they found that LL genotype and L allele were significant risk factors in the nonsmoker group [16]. Accordingly to this study, Malin et al. [17] also have found that in nonsmoking men the LL genotype could represent a genetic risk factor for carotid artery atherosclerotic disease. Unlike previous studies, Robertson et al. [18] found an higher CHD risk for LL smokers compared to LL nonsmokers. Although in our study we have not found an association between smoking habit and PON1 55 polymorphism, in accordance with the former studies we have found that patients carrying LL genotype have to face with a more seriously ACS episode. A study in which the PON1 55/192 haplotype was correlated with the number of diseased vessels emerged that subjects with PON1 RR/LL genotype possess an higher number of diseased vessels in respect to the other genetic variants [19]. The presence of LL genotype was also demonstrated to be associated with a more severe degree of IR (insulin resistance), suggesting that the IR might be the possible missing link between Met-Leu 55 PON polymorphism and the increased cardiovascular risk [20]. Accordingly, the 55 Leu allele was found to be associated with coronary artery disease only in the subgroup with metabolic syndrome [21]. Moreover, another paper reported that the LL homozygous men had more atherosclerotic plaques and complicated lesions in the common iliac arteries than the M allele carriers [22]. Conversely, in an Italian study the PON1 55 polymorphism was not found to be associated with carotid abnormalities [23].

An important limitation of this study is the lack of paraoxonase activity, as this could be useful to clarify the role of this enzyme in CHD and to remove the existing bulk of conflicting results.

Finally, our study suggests that PON1 55 polymorphism could play a role in the pathogenesis of cardiac ischemic damage. Hence, the significant association between PON1 55 LL polymorphism and the occurrence of a NSTEMI may contribute to improve the stratification of the cardiovascular risk in UA, NSTEMI, and STEMI being useful in providing a more accurate assessment of a patient prognosis.

## Figures and Tables

**Table 1:** Baseline clinical and genetics characteristics of 440 elderly ACS patients.

Characteristics	ACS patients (*N* = 440)	
	*N*	%
ACS diagnosis: UA (unstable angina)	98	22.3
ACS diagnosis: NSTEMI (no ST-elevation myocardial infarction)	135	30.7
ACS Diagnosis: STEMI (ST-elevation myocardial infarction)	207	47.0
Low HDL-cholesterol	133	30.2
No low HDL-cholesterol	307	69.8
Smokers	66	14.9
noncurrent smoker	374	85.1
History of CHD	288	65.4
No history of CHD	152	34.6
Type 2 diabetes mellitus	156	35.5
No Type 2 diabetes mellitus	284	64.5
Arterial hypertension	315	71.6
No arterial hypertension	125	28.4
Hypercolesterolemia	276	62.7
No hypercolesterolemia	164	37.3
Dead	80	27.0
Alive	216	73.0

**Table 2:** PON1 55 carriers frequencies of 440 ACS patients.

ACS diagnosis	M− (LL) *n* (%)	M+ (LM + MM) *n* (%)
UA (unstable angina)	36 (36.7)	62 (63.3)
NSTEMI (No ST-elevation myocardial infarction)	70 (51.9)	65 (48.1)
STEMI (ST-elevation myocardial infarction)	85 (41.1)	122 (58.9)

Total	191 (43.4)	249 (56.6)

*χ* ^2^ = 6.158, d.f. = 2, *P* = 0.046: for comparison between ACS diagnosis and PON55 carriers.

**Table 3:** Levels of biochemical parameters according to PON1 55 carriers in ACS patients.

Biochemical parameters	PON1 55 M− carriers (LL)	PON1 55 M+ carriers (LM + MM)	*P*
CK-MB (*μ*g/L) mean ± SD	78, 54 ± 118, 98	64, 76 ± 106, 22	0.273
Myoglobin (nmol/L) mean ± SD	51.49 ± 108.57	33.76 ± 67.99	**0.044**
Troponin I (*μ*g/L) mean ± SD	38, 38 ± 65, 36	26, 29 ± 61	0.118
C-reactive protein (nmol/L) mean ± S.D	62.19 ± 126.86	45.72 ± 58.10	0.728
